# Contribution of a Genomic Accessory Region Encoding a Putative Cellobiose Phosphotransferase System to Virulence of *Streptococcus pneumoniae*

**DOI:** 10.1371/journal.pone.0032385

**Published:** 2012-02-21

**Authors:** Lauren J. McAllister, Abiodun D. Ogunniyi, Uwe H. Stroeher, James C. Paton

**Affiliations:** Research Centre for Infectious Diseases, School of Molecular and Biomedical Science, University of Adelaide, Adelaide, South Australia, Australia; Indian Institute of Science, India

## Abstract

*Streptococcus pneumoniae* (the pneumococcus) is a formidable human pathogen, responsible for massive global morbidity and mortality. The ability to utilize carbohydrates in a variety of host niches appears to be integral to pneumococcal pathogenesis. In this study we investigated a genomic island, which includes a ROK family protein, a putative cellobiose phosphotransferase system (PTS) and a putative sulfatase. This accessory region is widespread in the pneumococcus in strains of various serotypes and levels of virulence. We have performed simple bioinformatic analysis of the region and investigated its role *in vivo* in 2 strains with markedly different virulence profiles (WCH206 of serotype 3, ST180; Menzies5 of serotype 11A, ST662). Deleting and replacing the entire island with an antibiotic resistance cassette caused the virulent serotype 3 strain to become attenuated in a murine pneumonia/sepsis model. Further mutants were constructed and used to show that various components of the island contribute significantly to the fitness of WCH206 in a variety of niches of this model, including the nasopharynx, ears and blood, but especially in the lungs. In addition, the island conferred a competitive advantage in nasopharyngeal colonization for the serotype 11A strain, which was essentially avirulent in the pneumonia/sepsis model. The contribution of this island to both pathogenesis and colonization may explain why this accessory region is widespread in the pneumococcus.

## Introduction

*Streptococcus pneumoniae* (the pneumococcus) is a formidable pathogen, which is the principal bacterial cause of pneumonia, sepsis and otitis media (OM), and responsible for more deaths worldwide than any other bacterium. The polysaccharide capsule is a *sine qua non* of virulence and defines the serotype of which there are over 90 identified to date. Nevertheless, additional non-capsular factors also play an integral role in pathogenesis. *S. pneumoniae* carries a large number of sugar transporters, which have been proposed to help it occupy unique *in vivo* micro-environmental niches [1]. However, little is known about pneumococcal carbohydrate utilization and metabolism in these environments [2]. Sugar transporters have been identified during signature-tagged mutagenesis virulence factor screens [3], [4], [5], [6], and several defined transporter mutants have been shown to be attenuated *in vivo* [2], [7]. This indicates that despite the large number of sugar transporters, the removal of even one can have a measurable impact on virulence.

**Figure 1:**
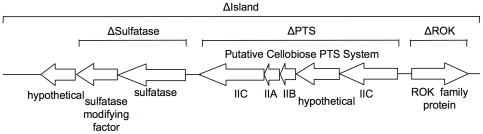
Organisation of the PTS island and location of mutagenesis deletions. Genes sph1922/spnoxc15810 – sph1930/spnoxc15890 of *S. pneumoniae* Hungary 19A-6 and OXC141 (serotype 3, ST180), which are present in our strains WCH206 and Menzies5, are represented. The locations of the various deletion mutants constructed in this study are indicated.

Some systems appear to be functionally conserved in the pneumococcus, such as the well-characterized neuraminidase NanA or the Sus and Scr sucrose utilization systems, although there can be sequence variation between strains [2], [8]. There are also carbohydrate utilisation systems encoded on accessory regions that are not uniformly distributed amongst strains [1], [9], [10], [11], [12]. McKessar and Hakenbeck have previously described an accessory region (the *cel* locus) containing a cellobiose phosphotransferase system (PTS) [13], whose components were also identified during a signature-tagged mutagenesis study using an otitis media model [6]. In this study we examined an alternative accessory region harbouring a putative cellobiose phosphotransferase system (PTS), which has been previously described as being absent in D39 and TIGR4 and hypothesised to be involved in virulence [14], [15], [16], [17], [18]. Despite its absence in D39 and TIGR4, this PTS was present in four distinct serotype 3 isolates (Menzies11 and Menzies17 of ST458, and WCH206 and WCH207 of ST180), which were highly virulent in a mouse sepsis model, but absent in the significantly less virulent serotype 3 strain WU2 (ST378) [18]. However, as with the *cel* locus, we found that this system is present in a wide range of pneumococcal isolates belonging to diverse serotypes (over 80% of those tested), including unrelated avirulent isolates of serogroup 11 [18]. The PTS is part of a larger 10 kb island (see Fig. 1), which also encodes a ROK family protein, a putative sulfatase (arylsulfatase) and a putative sulfatase modifying factor, as well as a couple of hypothetical proteins [14], [16], [17], [18]. The ROK family comprises transcriptional repressors, ORFs of unknown function and sugar kinases in bacteria, and includes a xylose repressor from *Bacillus subtilis*, a *N*-acetylglucosamine [GlcNAc] repressor, *nagC*, from *Escherichia coli*, and a fructose kinase from *S. mutans* [19]. In contrast, sulfatases are highly conserved across eukaryotes and prokaryotes and are believed to be used by prokaryotes to hydrolyse sulphate ester groups from proteoglycans, glycosaminoglycans (GAGs) and choline sulphate [20]. They are normally associated with a sulfatase-modifying factor [21], which is required to catalyse the post-translational activation of the sulfatase, an unusual feature for prokaryotic proteins [22]. There are many other pneumococcal sugar transporters also associated with hydrolases, but this particular system is interesting in that some strains, including G54 and serotype 3 isolates of ST458 (Menzies11 and Menzies17), have a deletion removing the sulfatase, as well as parts of the sulfatase modifying factor and the last gene of the PTS operon [18]. Due to the prevalence of this island and its interesting annotation, in this study we have constructed mutants of the island in two distinct *S. pneumoniae* genetic backgrounds and investigated the impact on phenotype *in vivo*.

## Results

### Bioinformatic analysis

We commenced by performing simple bioinformatic analysis of the various components of the AR. We observed that the ROK family protein does not appear to have the characteristic binding motif described by Titgemeyer et al. (1994), yet its size suggests it is more likely to be a repressor than a kinase [19]. A BLAST-p search revealed a NagC domain, but no DNA binding motif. In contrast, when the amino acid sequence of NagC from *E. coli* K12 (NC_000913) was examined, a MarR (repressor of the multiple drug resistance operon) family motif was detectable at the N-terminus. Interestingly, a Pfam search using the same sequences identified a MarR family motif in both proteins at the N-terminus, which was a significant match for NagC, but insignificant for the pneumococcal ROK family protein. HHPred indicated there is helix-turn-helix domain at the N-terminus of the pneumococcal ROK family protein and the best 3 matches, which had probabilities of 100 and E and P values of 0, were the well-characterised Mlc repressor of *E. coli*, which is homologous to NagC, an Mlc homolog of *Vibrio cholerae* and a putative N-acetylmannosamine repressor of *Thermotoga maritima* (also annotated as N-acetylglucosamine kinase). Interestingly, Pfam searches using the amino acid sequences of these 3 matches identified insignificant matches for the iron dependent repressor family, the MarR family and the MarR 2 family, respectively.

A BLAST-p search of various components of the putative cellobiose PTS revealed it belongs to the Lactose-N,N′-Diacetylchitobiose β-glucoside (Lac) family (Family 4.A.3), which is a family of β-glucoside, cellobiose and chitin disaccharide (N, N′-diacetylchitobiose) transporters. The hypothetical protein associated with the PTS components has been annotated as PEP phosphonomutase-like protein in some pneumococci. A HHpred search revealed the highest homology with inosine-5′-monophosphate dehydrogenase, GMP reductase 2 and most interestingly, N-acetylmannosamine-6-phosphate 2-epimerase 2 (probabilities greater than 99%).

Finally, HHPred analysis of the sulfatases identified homology (probability of 100, P and E values of 0) with other sulfatases as expected, as well as a phosphonate monoester hydrolase (from *Rhizobium leguminosarum*) and an alkaline phosphatase (from Antarctic bacterium TAB5). Interestingly, the hypothetical protein encoded adjacent to the sulfatase-modifying factor has a significant Pfam match with the TauE family (sulfite exporter TauE/SafE).

### Mutational analysis

To examine the phenotypic contribution of the genomic island encoding the putative cellobiose PTS, an in-frame, deletion-replacement mutant in the serotype 3 OM isolate WCH206 (ST180), designated WCH206 ΔIsland, was constructed by removing and replacing the entire island with an erythromycin resistance cassette attached to the promoter of the serotype 2 capsule locus, as described in the Materials and Methods and shown in **Figure 1**. This erythromycin resistance cassette has been previously used to construct nonpolar mutants of the capsular biosynthesis locus in D39 [23]. The orientation of the genes of the accessory region necessitated the inclusion of a promoter to allow expression of the erythromycin resistance gene, which is why the cassette was amplified from the mutant D39-ABΔe [23] to include the capsule locus promoter. Following confirmation of the mutation by sequencing, opaque variants were selected for all experiments, except the biofilm assay and colonisation competition study for which transparent pneumococci were preferred.

**Figure 2:**
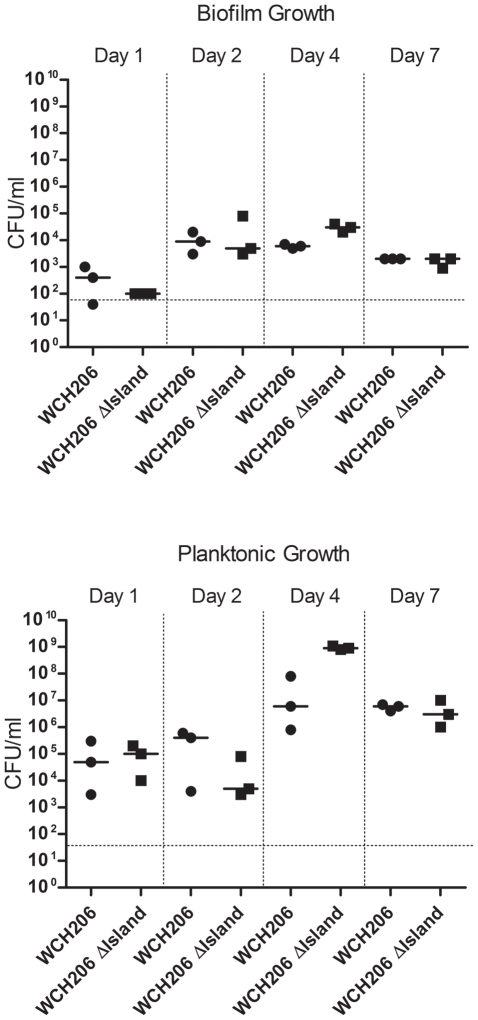
*In vitro* fitness of WCH206 ΔIsland. Biofilm and planktonic growth of WCH206 and WCH206 ΔIsland in 0.5×BHI was measured over a 7 day period. Median bacterial numbers are indicated by black, horizontal bars.

Initially, the mutant was examined for alterations in both planktonic and biofilm growth, as well as sugar fermentation. The numbers of biofilm-forming and planktonic bacteria were determined for WCH206 and WCH206 ΔIsland on days 1, 2, 4 and 7 in triplicate samples grown in 0.5×BHI. No significant differences in the growth characteristics or numbers of planktonic or biofilm-associated bacteria were observed at the time points examined (**Figure 2**). Fermentation of cellobiose by WCH206 and WCH206 ΔIsland grown in THY to mid-exponential phase was also investigated in two independent experiments, using the purple broth assay with sucrose as the positive control (see Materials and Methods). There was no difference between wild-type and mutant in their capacity to ferment cellobiose, although the colour change observed was less than that for sucrose (data not shown). Shafeeq et al. (2011) used M17 media to investigate the *cel* locus [24]. We cultured the mutant and wild-type in M17 supplemented with 5% (w/v) glucose, cellobiose or lactose. The previous observation that the PTS belongs to the Lactose-N, N′- diacetylchitobiose β-glucoside (Lac) family was the reasoning behind the inclusion of lactose. Although removing the island did not retard growth in these media (data not shown), growth in the cellobiose supplemented media was very poor compared to the other two media and neither strain grew over A_600_ 0.3 in the cellobiose supplemented media when the culture was started from BA. In addition to the growth assays, a sulfatase assay was performed using the WCH206 and WCH206 ΔIsland strains with 4-methylumbelliferyl sulfate as the substrate and limpet sulfatase as the positive control [25]. However, no activity was detectable under the conditions used in this study, even when undiluted lysate was used. The limpet sulfatase was still capable of cleaving 4-methylumbelliferyl sulfate when added to lysate of WCH206 suggesting there were no inhibitors of sulfatase activity present in the lysate.

**Figure 3:**
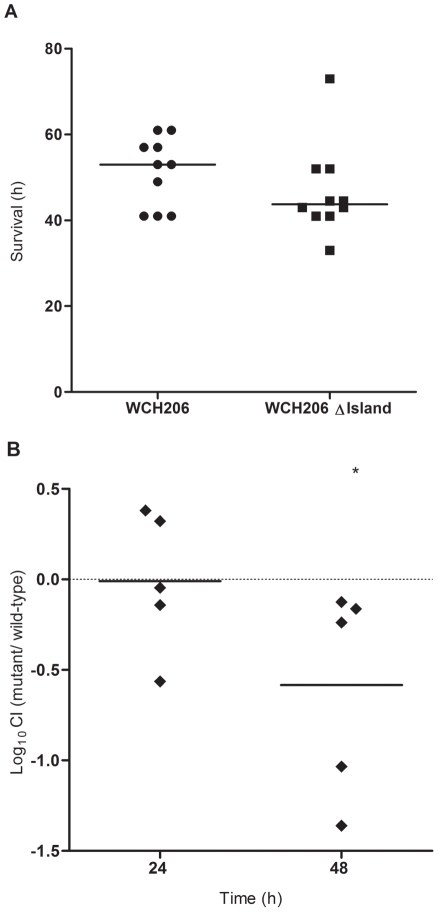
Fitness of WCH206 ΔIsland in the i.p. mouse model. (A) CD-1 mice were challenged i.p. with approximately 2.0×10^4^ CFU/mouse of either WCH206 or WCH206 ΔIsland. Median survival times are indicated by black, horizontal bars and were compared using the Mann Whitney *U* test (two tailed); NS, not significant. (B) CD-1 mice were challenged i.p. with 2×10^3^ CFU/mouse of a mixture of WCH206 and WCH206 ΔIsland at a ratio of approximately 1∶1.7. Mice were sacrificed at 24 h and 48 h post-challenge and the ratio of wild-type to mutant calculated to determine the log_10_CI. The neutral value of 0 (mutant is as virulent as the wild-type) is indicated by a broken horizontal line. Log_10_CIs less than 0 indicates that the mutant is less fit in comparison to the wild-type. The mean log_10_CIs are indicated by black, horizontal bars. One sample *t*-tests were used to compare the mean log_10_CI of each group to the hypothetical value of 0: *, *P*<0.05.

**Figure 4:**
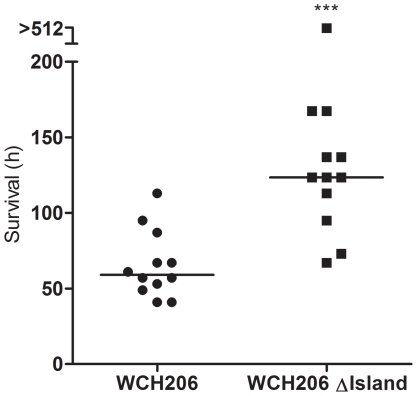
Fitness of WCH206 ΔIsland in the pneumonia/sepsis model. CD-1 mice were challenged i.n. under anaesthesia with approximately 1.2×10^7^ CFU/mouse of either WCH206 or WCH206 ΔIsland. Median survival times are indicated by black, horizontal bars and were compared using the Mann Whitney *U* test (two tailed): ***, *P*<0.001.

Effects on virulence were then assessed using i.p. and pneumonia/sepsis murine models. In a direct i.p. (sepsis) challenge model, the median survival time of mice challenged with WCH206 ΔIsland was not significantly different from that for mice challenged with the otherwise isogenic wild-type strain (**Figure 3A**). As subtle differences in virulence could be masked due to host-to-host variation, we then compared the mutant and wild-type in a mixed infection model (i.p. competition), and found the mutant was significantly attenuated at 48 h post-infection (*P* = 0.0252) (**Figure 3B**). In the pneumonia/sepsis model, where mice were inoculated intranasally (i.n.) under anaesthetic, there was a significant increase in the median survival time for mice infected with the WCH206 ΔIsland mutant compared to the wild-type (*P*<0.001) (**Figure 4**). Taken together, the results from the i.p and i.n. studies suggest that the genomic island encoding the PTS system plays a significant role in pneumonia and sepsis.

**Figure 5:**
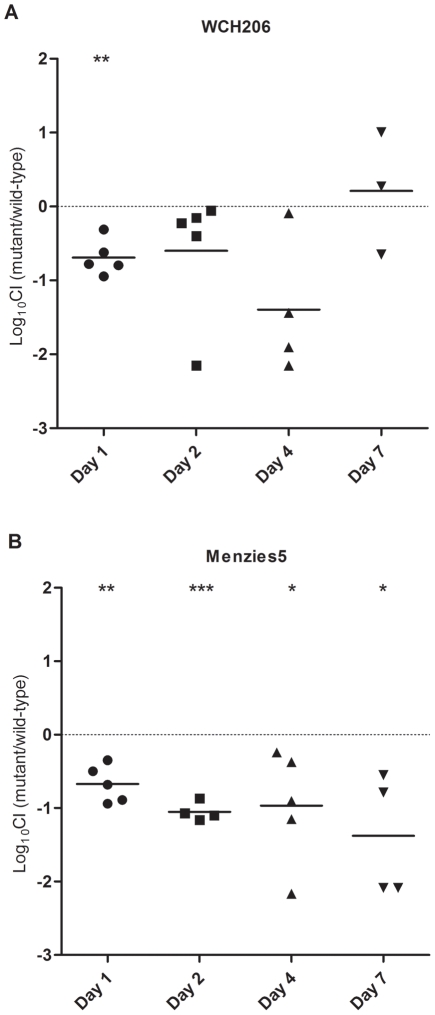
Fitness of ΔIsland in the nasopharynx over a 7 day period. CD-1 mice were inoculated i.n. without anaesthesia with 2×10^7^ CFU/mouse of a combination of WCH206 and WCH206 ΔIsland (serotype 3) at a ratio of 1∶4 (panel A) or 1.5×10^6^ CFU/ml of a combination of Menzies5 and Menzies5 ΔIsland at a ratio of 9∶7 (panel B). The log_10_ CIs were calculated on days 1, 2, 4 and 7 for the nasopharynx (nasopharyngeal tissue and wash combined). The neutral value (0) is indicated by a broken, horizontal line and the mean log_10_ CIs are indicated by black, horizontal bars. Log_10_ CI values under 0 indicate that the mutant is less fit than the respective wild-type. One sample *t*-tests were used to compare the mean log_10_ CI of each group to 0: *, *P*<0.05; **, *P*<0.01; ***, *P*<0.001.

The ability of WCH206 ΔIsland to colonize the nasopharynx of mice was also examined. Mice were inoculated intranasally without anaesthesia and the nasopharynx was washed and nasopharyngeal tissue removed and homogenized 1, 2, 4 and 7 days post-inoculation. Data from the nasopharyngeal wash and homogenate were combined. The mutant was found to be significantly attenuated compared to wild-type WCH206 strain on day 1; log_10_CI values were less than 0 on days 2 and 4 as well, but this did not reach statistical significance (**Figure 5A**). As serogroup 11 strains had previously been found to colonize at higher numbers on days 1 and 2 post-inoculation than serotype 3 strains in this model [18], we also constructed an island deletion replacement mutant in the serotype 11A carriage isolate Menzies5 (ST662) using the same method. Replacing the entire island with an erythromycin cassette downstream of a capsule locus promoter also did not affect *in vitro* growth of this strain, including in M17 medium supplemented with lactose and cellobiose, although both strains grew poorly in the presence of cellobiose (data not shown). Infection with a mixed culture (Menzies5 and Menzies5 ΔIsland) was performed and the competitive indices in the nasopharynx calculated. The mutant was significantly attenuated compared to the wild-type at all time points for Menzies5 (**Figure 5B**).

Since the mutants described above had deletions of the entire island, we sought to determine which individual components of this region were responsible for the attenuation. For this purpose, non-polar deletion replacement mutants of the various components were constructed using the previously described erythromycin resistance cassette without the inclusion of the D39 capsule locus promoter. The first mutation was a deletion of the ROK family protein, the second was of the putative cellobiose PTS and associated hypothetical protein, and the third was of the sulfatase and the sulfatase modifying factor genes (see **Figure 1**). Mutants were constructed for both Menzies5 and WCH206, but the mutants in Menzies5 were not ultimately used for any animal work. However, due to the low transformability of WCH206 and the conservation of the genes in the accessory region and flanking areas, the erythromycin replacement mutations were amplified from the Menzies5 mutants and introduced to competent WCH206 cells rather than the respective overlap PCR product. Following successful transformation, the erythromycin replacement mutation was subsequently amplified from the WCH206 mutant and introduced to a fresh aliquot of WCH206 competent cells to minimize the co-transformation of non-contiguous chromosomal DNA. The second round transformant was then confirmed by sequencing.

Opaque variants were required for the animal model. However, there were issues in obtaining opaque for some mutants *in vitro*. Therefore, a mouse passage was performed to facilitate the isolation of opaque variants. All mutants and the wild-type strain were similarly passaged for consistency.

**Figure 6:**
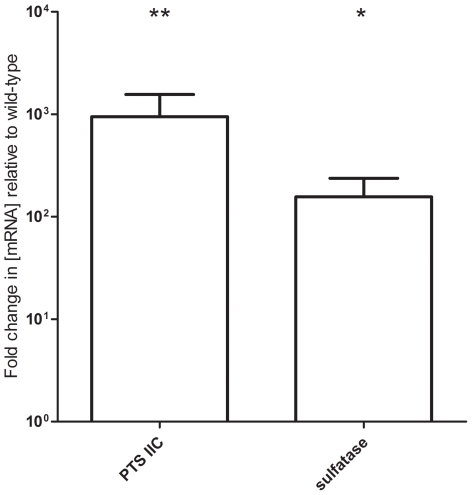
*In vitro* expression of the putative cellobiose PTS and sulfatase genes in WCH206 ΔROK. Data were calculated using the 2^−ΔΔCT^ method with 16s rRNA acting as an internal control and represent the fold difference between [mRNA] in WCH206 ΔROK relative to WCH206. Error bars represent the 95% confidence interval. In addition, the fold difference in [mRNA] of the first PTS IIC component and sulfatase (annotated as CGSSp3BS71_03497 and CGSSpBS71_3467 in Sp3-BS71 [NCBI RefSeq: ZP_01817543.1]) in relation to 16s rRNA (internal control) was calculated and compared between mutant and wild-type using an unpaired *t*-test (*, *P*<0.05; **, *P*<0.01).

**Figure 7:**
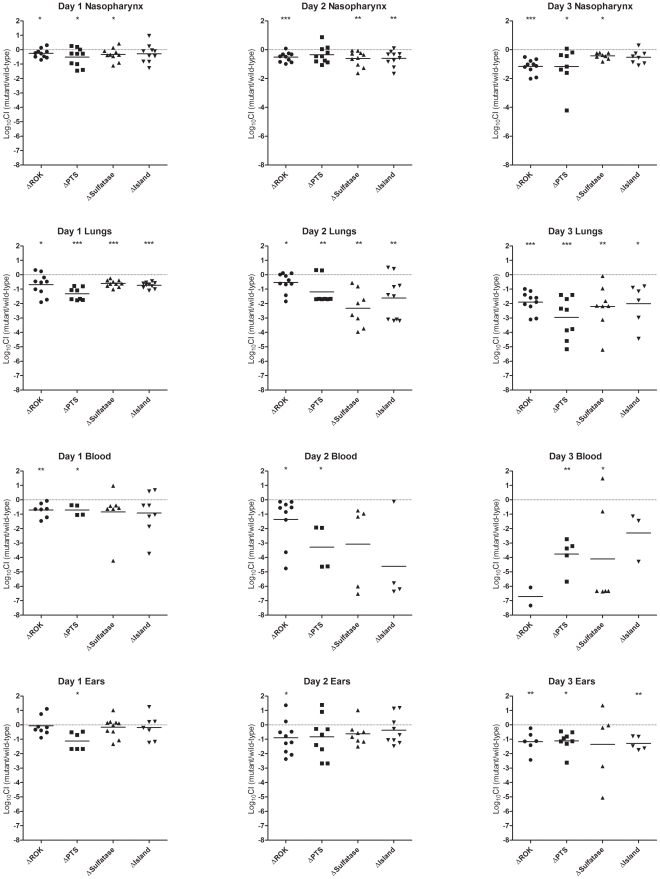
Competitiveness of various Island mutants in the pneumonia/sepsis model. Data are combined from duplicate experiments. Mice were inoculated with approximately 3×10^7^ CFU/mouse of a combination of WCH206 and either WCH206 ΔROK, ΔPTS, ΔSulfatase or ΔIsland, at average input ratios of 1.75, 0.5, 1.65 and 1.45, respectively. The CIs were calculated on days 1, 2 and 3 for the nasopharynx (nasopharyngeal tissue and wash combined), lungs, blood and ears, and the mean log_10_ CIs are indicated by black, horizontal bars. The neutral value is 0 (indicated by a broken, horizontal line). One sample *t*-tests were used to analyze the data (hypothetical value = 0): *, *P*<0.05, **, *P*<0.01, ***, *P*<0.001.

These defined mutants of WCH206 showed no alteration in their *in vitro* growth characteristics (data not shown). However, examination of the transcription activity of genes in this region revealed that the transcripts of the sulfatase and first IIC component of the putative cellobiose PTS were increased significantly in the ΔROK mutant (**Figure 6**), although sulfatase activity was also not detectable for this mutant. The mutants were subsequently assessed in a pneumonia/sepsis competition model with the isogenic wild-type strain at 24, 48 and 72 h post-infection. In the nasopharynx, the mean log_10_CI was below 0 for every mutant at each time point, which was statistically significant for ΔROK and ΔSulfatase on all days, for ΔPTS on days 1 and 3, and for ΔIsland on day 2 (**Figure 7**). Furthermore, significant attenuation for all mutants was observed in the lungs at all time points (**Figure 7**). In the blood, where there is a high degree of bacteremia, the ΔPTS mutant was significantly attenuated at all time points; the ΔROK mutant was significantly attenuated on days 1 and 2, while the ΔSulfatase strain was significantly attenuated on day 3 (**Figure 7**). In the ears, mutants typically displayed significant attenuation by day 3. The ΔSulfatase mutant did not show statistically significant attenuation on day 3, probably because only half of the mice had bacteria isolated from their ears at this time point, and there was a wide spread in the log_10_CI values (**Figure 6**).

## Discussion

Previous studies have indicated the importance of sugar transporters in pneumococcal virulence [2], [3], [4], [5], [6], [7]. In this study an accessory region including a putative cellobiose PTS, a putative sulfatase and ROK family protein has been investigated. This accessory region is widely distributed, but was found to be one of the distinguishing genetic features between highly virulent strains of serotype 3 and the less virulent serotype 3 strain WU2 [18]. In the present study, we have examined the fitness of PTS island mutants in various *in vivo* models. These studies were carried out using mutants of two unrelated type 3 (ST180) and type 11A (ST662) clinical isolates, with markedly different virulence profiles. Initially the entire island was replaced with an erythromycin resistance cassette in the serotype 3 strain WCH206 (ST180), which is highly virulent in our i.p. and pneumonia/sepsis models. In this background, deleting the island did not have a negative impact on the ability to grow or form biofilms in standard laboratory media. We also could not demonstrate differences in the ability to ferment cellobiose or to grow in cellobiose-supplemented M17 medium, as well as to grow in lactose-supplemented medium, although we found growth and fermentation to be poor in the presence of cellobiose in contrast to other sugars. Lack of a sugar uptake defect phenotype could be attributable to the presence of other complementary PTSs, such as that described by McKessar and Hakenbeck [13]. Similarly, a biochemical phenotype could not be assigned to the putative sulfatase mutant.

As the ΔIsland mutant did not demonstrate any in vitro growth defects, the behaviour of the mutant and wild-type were subsequently examined *in vivo*. When WCH206 and WCH206 ΔIsland were compared in two virulence models, survival time increased significantly for mice inoculated with the mutant in the pneumonia-sepsis model but not the i.p. model (**Figures 3A** **and** **4**). However, when the mutant and wild-type were tested in the more sensitive i.p. competition model, the mutant was isolated less frequently from the blood 2 days after inoculation (**Figure 3B**). In addition to virulence, colonization was also investigated. A ΔIsland mutant of the low-virulence serotype 11A strain Menzies5 (ST662) was also examined, as serogroup 11 strains are better colonizers than type 3 in the i.n. model [18]. WCH206 ΔIsland was attenuated significantly in the nasopharynx on day 1 relative to the wild-type; log_10_CI values were less than 0 on days 2 and 4 as well, but this did not reach statistical significance (**Figure 5A**). However, in the Menzies5 background, the mutant was significantly less fit in the nasopharynx at every time point. These results suggest that the island plays a significant role in colonization of both strains and invasive disease caused by WCH206 (**Figure 5B**).

In an attempt to attribute the virulence defect of ΔIsland mutants to individual genes, mutants of the ROK family protein, PTS operon and sulfatase/sulfatase modifying factor were constructed in WCH206. Again there was no impact on growth, but removing the ROK family protein resulted in increased sulfatase and PTS transcription. Bioinformatic analysis of the ROK family protein had previously suggested it was more likely to be a repressor than a kinase. These results are consistent with the recent demonstration that the role of the transcriptional regulator CelR encoded on the pneumococcal *cel* locus (another accessory region), is to activate expression of both the cellobiose PTS and 6-phospho-β-glucosidase of this locus [24]. In our study, the impact on the respective protein levels is not known and it may be that there is also post-translational control of expression. The loss of the PTS and sulfatase presumably impacts the ability of WCH206 to utilize their substrate(s). However, carbon catabolite control is essential to the fitness of bacteria, as carbohydrates must be utilized in preferred order for optimal growth. Therefore, loss of repression of these genes could also be detrimental to fitness. Previously, loss of the pneumococcal lactose-inducible β-galactosidase repressor RegM (also known as catabolite control protein A [CcpA]) has been shown to result in decreased fitness *in vivo* [26], [27].

A 3 day competitive study was conducted with the various WCH206 mutants using the pneumonia/sepsis model and the results suggested the island is important in a variety of niches, particularly the lungs, where there was statistically significant attenuation for all mutants at each time point (**Figure 7**). Attenuation was observed for 3 out of 4 mutants in the nasopharynx over the 3 day period, adding further evidence for the island playing a role in nasopharyngeal fitness. Furthermore, this accessory region may also play a role in otitis media, as the majority of mutants were significantly attenuated in the ears by day 3. With regard to the impact of the various mutations on development of bacteremia, it is possible that diminished ability to colonize lung tissue could limit translocation into the blood and hence the level of bacteremia. Nevertheless, the i.p. competition study indicates that the island has an additional impact on sepsis over and above lung colonization and lung-blood translocation. This could not be correlated with survival, however, as mice inoculated i.p. with WCH206 ΔIsland did not survive significantly longer than those challenged with wild-type (**Figure 3A**). Overall, the pneumonia/sepsis competition study demonstrates that multiple components of the island contribute to fitness in the various niches. This includes the putative sulfatase, even though there are some *S. pneumoniae* strains, which carry a variant of the island with a deletion in the putative sulfatase. Unfortunately, our ST458 isolates with this deletion were not transformable in vitro, so we were unable to assess the impact in this genetic background. It is possible that there is another hydrolase compensating for the loss of the putative sulfatase or the incomplete accessory region still gives a competitive advantage in these particular strains.

The results of this study show that despite the large number of sugar transporters in the pneumococcal genome, the removal of even one sugar utilization system can have an impact on virulence. Little is known about the *in vivo* substrates of many of the putative pneumococcal sugar transporters, although the hydrolases of two putative pneumococcal fucose operons have been recently shown to target different blood group antigens [28]. The competitive advantage conferred by the PTS island in a variety of niches suggests the substrates of the putative cellobiose PTS and putative sulfatase of this study are present in multiple *in vivo* niches. The bioinformatic analysis conducted in this study adds further weight to the likely function of the PTS as a sugar transporter. While it is yet to be determined whether the PTS and putative sulfatase work together, many host sugars undergo modifications, such as sulfation [29], [30], [31]. It is interesting that the PTS belongs to the chitin disaccharide transporter family, as although chitin is found in the outer covering of insects and crustaceans, it is made of GlcNAc [32]. The homology of the ROK family protein to a putative N-acetylmannosamine repressor and that of the hypothetical protein associated with the PTS components to N-acetylmannosamine-6-phosphate 2-epimerase is also of interest.

Overall, the results of this study show that the pneumococcal genomic island encoding the putative cellobiose PTS confers a competitive advantage in both passive colonization and active invasion models. This is not unexpected given that this region is found in a wide variety of pneumococcal serotypes and STs, including isolates with markedly differing virulence profiles. However, the PTS island is not essential for virulence, and as the island is also found in avirulent strains, the importance of the system in pathogenesis ultimately depends on the strain background. Thus, accumulation of multiple beneficial accessory regions, such as the one described in this study, rather than carriage of any single specific region, is likely to determine the human virulence characteristics of *S. pneumoniae* isolates.

## Materials and Methods

### Ethics statement

All animal experiments were approved by the Animal Ethics Committee of the University of Adelaide (Project Number: S- 86-2006), and complied with the Australian code of practice for the care and use of animals for scientific purposes (7th Edition 2004) and the South Australian Animal Welfare Act 1985. Written consent was obtained for studies of human specimens and ethics approval was obtained from the Human Research Ethics Committe of the Menzies School of Health Research and Department of Health and Families.

### Bacterial strains and growth conditions

The middle ear isolate WCH206 (serotype 3, ST180), nasopharyngeal isolate Menzies5 (serotype 11A, ST662) and the mutant D39-ABΔe have been described previously [18], [23]. *S. pneumoniae* strains were grown in THY (Todd-Hewitt broth [Oxoid, Basingstoke, United Kingdom] supplemented with 1% Bacto™ yeast extract [Beckton, Dickinson and Company, Franklin Lakes, NJ, USA]), serum broth (10% [v/v] donor horse serum in nutrient broth), brain heart infusion (BHI) broth [Oxoid], M17 broth [Oxoid] supplemented with 5% (w/v) glucose (Ajax Finechem, Sydney, Australia), lactose (Ajax Finechem) or cellobiose (Sigma-Aldrich, St. Louis, MO, USA), or on blood agar (BA) plates at 37°C in 95% air and 5% CO_2_. Erythromycin (Ery; Roche, Mannheim, Germany), gentamicin (Sigma-Aldrich,) and optochin (Sigma-Aldrich,) were added at concentrations of 0.2 µg/ml, 5 µg/ml and 5 µg/ml, respectively, where appropriate. Pneumococci were transformed using complete transformation medium (CTM) [26], [33]. Sugar fermentation assays were carried out using BBL™ Purple Broth Base (Beckton, Dickinson and Company) essentially as previously described [13]. Static Biofilm assay was performed as described previously [34], except that 0.5×BHI was used in place of THY.

### DNA manipulations

Overlap extension PCR was used to construct mutants [35], [36]. The primers J214 and J215 [37] were used to amplify the erythromycin resistance gene (*ery*) from D39-ABΔe [23]. For ΔIsland mutants, Pcps F (5′GGTTCGCGGGAAGTCTACTAAG3′) was used in place of J215 to include the *cps*2 promoter. WCH206 ΔIsland and MSHR5 ΔIsland were constructed using the primers Pcps/sph1930/31 (5′CTTAGTAGACTTCCCGCGAACCTATCTCCCCTTTTTCACAA TGTATAG3′), sph1932 (5′CCCTATATCATGGCTGGTGACC3′), J215/sph1920/21 (5′CGGGAGGAAATAATTCTATGAGGAAAGCAAACAGCCTTGAAATCAAT) and sph1914 (5′CTAATTCAGTCCAAACTTCCAGTTC3′). WCH206 ΔROK was constructed using the primers J214/sph1930 (5′TTGTTCATGTAATCACTCCTTCTAG TTGTCAGATTCTTAAAATCCTAT3′), G54 spn02109 (5′CCCTGTGCCTCTCTTGT CAACA3′), J215/sph1930 (5′CGGGAGGAAATAATTCTATGAGACATTTAATAA ATAGGCTAAAAAGAGG3′) and sph1932 (5′CCCTATATCATGGCTGGTGACC3′). WCH206 ΔPTS was constructed using the primers J214/sph1929 (5′TTGTTCATGTAA TCACTCCTTCAAATATATTTTATTATCAAAA GTTATCAATTA3′), sph1930 (5′GTGCGGCTGTAAAATCATTGTAAG3′), J215/sph1925 (5′CGGGAGGAAATAA TTCTATGAGTTGCGTTTTTTGTGATAAAATAGAAATAGA3′) and sph1924a (5′GGGGATTTAATGGAGCATGTGGT3′). WCH206 ΔSulfatase was constructed using J214/sph1924 (5′TTGTTCATGTAATCACTCCTTCAATATGGCAAAAGAGG CACAAGAA 3′), mRNA sph1925 PTS IIC R (see below), J215/sph1923 (5′CGGGAG GAAATAATTCTATGAGAAAAAGTGGTTGAGATTATTTATTTT3′) and sph1919 (5′AGTTGCCGAAGACGCTAAAGACG3′). All primers were purchased from Sigma-Aldrich. PCR reactions were carried out using the Expand™ Long Template or High Fidelity PCR Systems (Roche Diagnostics) and an Eppendorf Mastercycler (Eppendorf, Hamburg, Germany). Mutations were confirmed by sequencing using the primers J257 (5′TTAAATGCCCTTTACCTGTTCC3′) and J258 (5′GAAGCTATATACGTACTTTG TTTC3′). DNA sequencing reactions were carried out using the BigDye® Terminator v3.1 Cycle Sequencing Kit (Applied Biosystems, California, USA).

### RNA analysis

Duplicate cultures in BHI were inoculated from BA plate cultures of each strain and grown to mid-exponential phase. RNA was isolated and purified from bacterial pellets with acid-phenol∶chloroform∶isoamyl alcohol (125∶24∶1; pH 4.5, Ambion, Austin, TX., USA) essentially as described previously [38], [39]. Differences in gene transcript levels was assessed by real-time RT-PCR on a Rotorgene RG-2000 (Corbett Research, Mortlake, NSW, Australia) using the Invitrogen SuperScript™ III Platinum® SYBR® Green One-Step qRT-PCR kit. A primer set specific for 16S rRNA (mRNA spd0016 16s F 5′GAGCTTGCTTCTCTGGATGAGTTG3′, mRNA spd0016 16s R 5′GTGATGCAAGTGCACCTTTTAAGC3′) was used as internal controls for data normalization. Transcripts of the cellobiose PTS were measured using mRNA sph1929 PTSIIC F (5′ACCTCCCAAAGCTACTTGAGCAAT3′) and mRNA sph1929 PTSIIC R (5′GATGGTGTTCCACCAGCAGTATCT3′). Transcripts of the sulfatase were measured using mRNA sph1924 sulfatase F (5′CAATAAAGCTGCCCTAGCAGGAA C3′) and mRNA sph1924 sulfatase R (5′GATCAAATGAGAGCAGACGCCTTA3′). Quantitative fold differences for each transcript were determined using the 2^−ΔΔ*C*T^ method [40].

### Virulence and pathogenesis studies

Each animal experiment used outbred 5–6 week old CD-1 (Swiss) mice and was approved by the Animal Ethics Committee of the University of Adelaide. For intraperitoneal (i.p.) and pneumonia/sepsis models, opaque variants were cultured in SB to approximately 1×10^8^ CFU/ml (A_600_ = 0.16), followed by dilution in sterile SB to the appropriate challenge dose [18], [41]. I.p challenge was performed by injecting mice i.p. with 100–200 µl of inoculum. Mice in the pneumonia/sepsis study were anesthetized with Nembutal (pentobarbitone sodium, Rhone-Merieux) and challenged intranasally (i.n.) with 50 µl of inoculum, as previously described [18], [38], [39]. The actual challenge doses were determined retrospectively by plating on BA with or without antibiotic selection, as appropriate. For survival studies, mice were monitored closely over a 3 week period following challenge for signs of disease and the survival time of each mouse was recorded. Inoculation of the mice in the i.p. and pneumonia/sepsis competition studies was performed as for the former studies, but a combination of wild-type (Ery^s^) and mutant (Ery^r^) was used. For the i.n. colonization competition study, transparent phase bacteria were grown in THY to mid-logarithmic phase (A_600_ = 0.5) [18], [41]. Mutant (Ery^r^) and wild-type (Ery^s^) cultures were then concentrated 10-fold, combined, and 10 µl was used to inoculate each mouse i.n. without anaesthesia. For all competition studies, the exact challenge doses and ratio of mutant to wild-type (input ratio) was determined retrospectively by duplicate plating on BA and BA-Ery. Mice were sacrificed by CO_2_ asphyxiation and nasal wash, ear wash, nasal tissue, lung, and blood samples were taken where appropriate and processed as previously described [18], [38], [39], [41]. Samples were plated onto BA and BA-Ery to determine the ratio of mutant to wild-type (output ratio) for each niche at each time point. Competitive Index (CI) was calculated as output ratio/input ratio.

### Sulfatase assay

The assay was conducted using 4-methylumbelliferyl sulfate potassium salt (Sigma-Aldrich) and limpet sulfatse (Sigma-Aldrich) as a positive control, as previously described [25], with the exception that bacteria were grown in BHI and lysed by either a French pressure cell (SLM Instruments, Urbana, IL, USA) operated at 12,000 psi or with 0.1% (w/v) sodium deoxycholate (BDH Biochemicals, Poole, United Kingdom).

### Bioinformatic analysis

In this study, the sequence of the accessory region encoding a putative cellobiose PTS was taken from either *Streptococcus pneumoniae* Hungary19A-6 (CP000936.1; nucleotides 1784680–1794642) or OXC141 (FQ312027.1; nucleotides 1602906–1612886). BLAST (basic local alignment search tool) analysis was performed through the websites of the National Center for Biotechnology Information (NCBI) and the Transporter Classification Database (TCDB) [42], [43]. Searches of the Pfam database (version 25.0) were also performed, as well as on the HHpred server [44], [45].
